# Case report: Successful treatment of a rare HER2-positive advanced breast squamous cell carcinoma

**DOI:** 10.3389/fphar.2024.1332574

**Published:** 2024-02-22

**Authors:** Gui Wang, Chenghui Yang, Donglin Zeng, Jihao Wang, Huaxin Mao, Yu Xu, Chao Jiang, Zhen Wang

**Affiliations:** ^1^ Department of General Surgery, Longquan People’s Hospital, Lishui, China; ^2^ Department of Breast Surgery, Second Affiliated Hospital, Zhejiang University School of Medicine, Hangzhou, China; ^3^ Department of Breast Surgery, First Affiliated Hospital of Wenzhou Medical University, Wenzhou, China; ^4^ Academy of Chinese Medical Sciences, Zhejiang Chinese Medical University, Hangzhou, China; ^5^ Key Laboratory of Tumor Microenvironment and Immune Therapy of Zhejiang Province, Second Affiliated Hospital, Zhejiang University School of Medicine, Hangzhou, China; ^6^ Cancer Center, Zhejiang University, Hangzhou, China

**Keywords:** metaplastic breast cancer, breast squamous cell carcinoma, HER2, targeted therapy, antibody drug conjugate

## Abstract

**Background:** Breast squamous cell carcinoma (SCC) is an uncommon and highly aggressive variant of metaplastic breast cancer. Despite its rarity, there is currently no consensus on treatment guidelines for this specific subtype. Previous studies have demonstrated that chemotherapy alone has limited efficacy in treating breast SCC. However, the potential for targeted therapy in combination with chemotherapy holds promise for future treatment options.

**Case presentation:** In this case report, we present a patient with advanced HER2-positive breast SCC, exhibiting a prominent breast mass, localized ulcers, and metastases in the lungs and brain. Our treatment approach involved the administration of HER2-targeted drugs in conjunction with paclitaxel, resulting in a sustained control of tumor growth.

**Conclusion:** This case represents a rare occurrence of HER2-positive breast SCC, with limited available data on the efficacy of previous HER2-targeted drugs in treating such patients. Our study presents the first application of HER2-targeted drugs in this particular case, offering novel therapeutic insights for future considerations. Additionally, it is imperative to conduct further investigations to assess the feasibility of treatment options in a larger cohort of patients.

## Background

Metaplastic breast cancer (MpBC) is a collection of morphologically diverse and exceedingly aggressive variant diseases with a low prevalence, comprising 0.2%–1.0% of the overall breast cancer cases (Pezzi et al., 2007; DeSantis et al., 2017; Thomas et al., 2023a). In the year 2000, the World Health Organization (WHO) officially classified MpBC as a distinct and heterogeneous form of invasive breast cancer, distinguished by the transformation of neoplastic epithelium into squamous epithelium and/or stromal elements, such as spindle cells, chondrocytes, and osteocytes (Thomas et al., 2023b). MpBC can be categorized into various histological subtypes, consisting of one or multiple components or a combination of carcinoma and metaplasia regions. Based on the pathological features, the histological grade of MpBC can be classified as high or low grade. High-grade MpBC encompasses squamous cell carcinoma (SCC) which accounting for about 0.1%–0.2% of breast cancer (Hennessy et al., 2005), spindle cell carcinoma, metaplastic carcinomas with mesenchymal differentiation (such as cartilage, bone, rhabdomyosarcoma, and nerve differentiation), as well as mixed metaplastic carcinomas. Low-grade MpBC encompasses low-grade adenosquamous carcinoma and fibromatosis-like metaplastic carcinomas. The majority of MpBC cases exhibit negative expression of estrogen receptor (ER), progesterone receptor (PR), and human epidermal growth factor receptor 2 (HER2), resembling triple-negative breast cancer (TNBC) but with a worse prognosis than conventional TNBC (Narayan et al., 2023; Tan et al., 2023; Zheng et al., 2023). In recent years, advancements in research technology have led to an enhanced understanding of this uncommon breast tumor, resulting in increased attention and the accumulation of relevant treatment experience for MpBC (Qiu et al., 2022; Corso et al., 2023; Coutant et al., 2023; Kawachi et al., 2023).

Herein, we report the initial case of a patient diagnosed with HER2-positive breast SCC, who achieved sustained tumor control through the utilization of HER2-targeted drugs in conjunction with paclitaxel.;

## Case description

**FIGURE 1:**
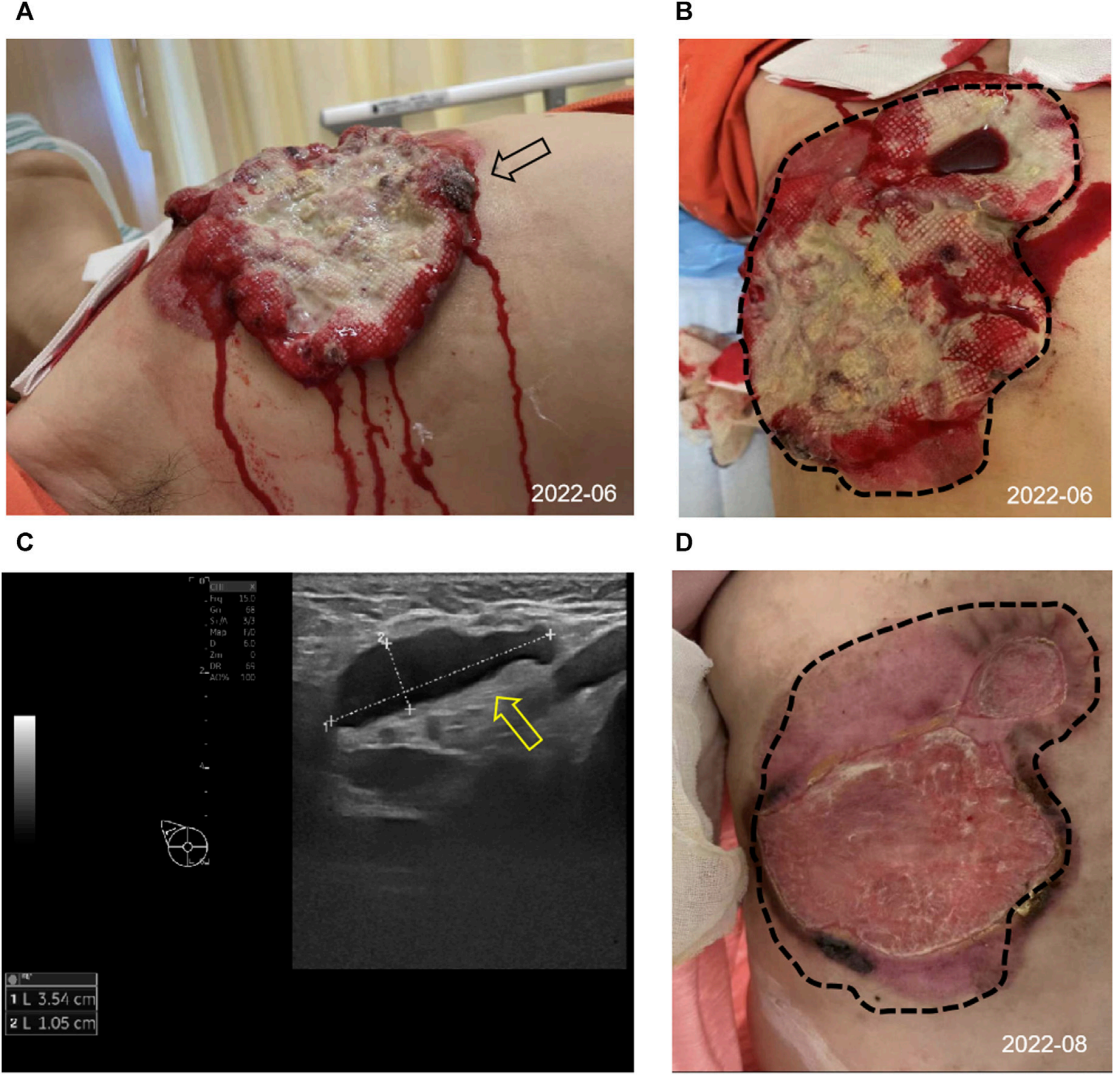
Appearance and clinical sign at initial diagnosis and after 2 cycles of treatment. **(A,B)** Image of the right chest mass from side (black arrow) and frontal (tumor lesions outlined with black dashed lines) at the initial diagnosis. **(C)** The ultrasound image showed enlarged right axillary node (3.54*1.05 cm) with disappearance of the normal lymph node structure (shown by the yellow arrow). **(D)** Changes in the right chest mass appearance after 2 cycles of treatment, compared with initial diagnosis (black dashed lines).

In June 2022, a 52-year-old female patient sought medical attention at the clinic, reporting the presence of a right breast mass persisting for a duration of 6 months, accompanied by local skin ulceration lasting over 3 months. The patient described the initial discovery of the breast mass as protruding through the skin, measuring approximately 2*2 cm in size and exhibiting a red coloration. Due to tingling pain, the patient engaged in repeated wiping of the mass, which unfortunately led to its gradual enlargement and subsequent rupture within a span of 2 months, resulting in the exudation of hemorrhagic fluid. Despite these concerning developments, the patient remained unaware of the severity of her condition and refrained from seeking medical attention. Subsequently, there was a significant escalation in the extent of skin ulceration. Upon physical examination, a breast mass exhibiting a concave alteration and surface hemorrhaging was observed, measuring approximately 15*10 cm (Figures 1A, B). Additionally, several enlarged and firm consistency right axillary lymph nodes were detected. The patient denied any familial history of breast cancer.

**FIGURE 2:**
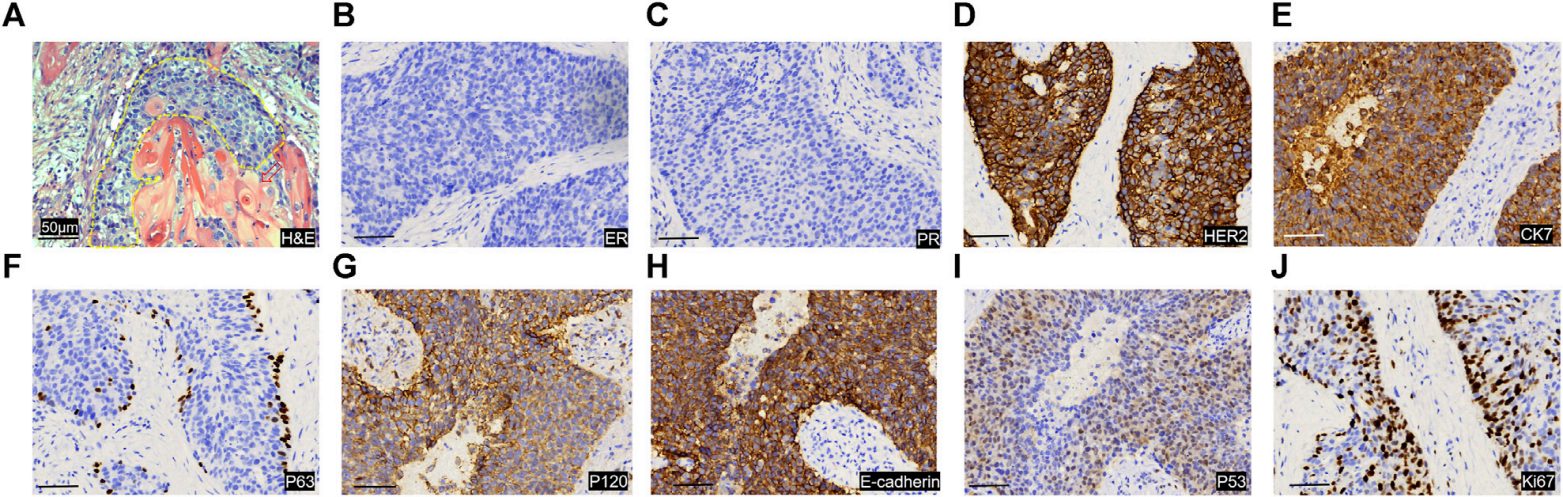
Image of pathological sections of tumor puncture. **(A)** H&E image of the pathological section. The yellow dashed line shows the tumor cancer foci, and the tumor cells grow in a nest, with obvious cell specificity, and nuclear division. Significant cell keratosis is seen at the positions indicated by the red arrows. Immunohistochemical staining images of for ER **(B)**, PR **(C)**, HER 2 **(D)**, CK7 **(E)**, P63 **(F)**, P120 **(G)**, E-cadherin **(H)**, P53 **(I)**, Ki-67 **(J)**. Scale Bar = 50 μm.

**FIGURE 3:**
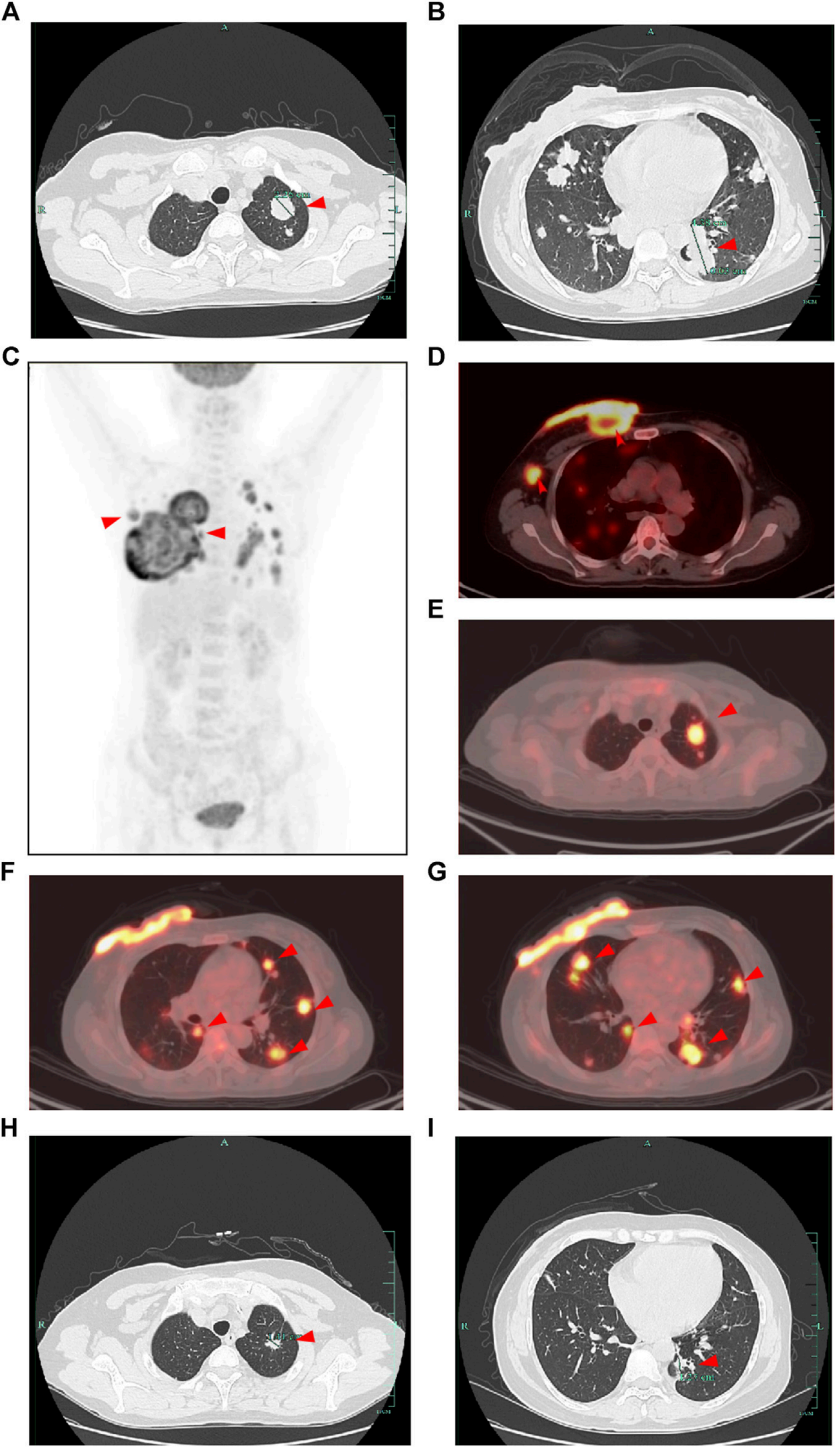
CT and PET-CT images of the patient. **(A,B)** CT images of the lung at the initial diagnosis showed multiple lung metastasis (shown by the red arrow). **(C)** Overview of the whole-body PET-CT image. **(D–G)** PET-CT image showed an increased uptake of fluorodeoxyglucose (FDG) in right chest wall soft tissue, right axillary lymph nodes, and multiple parts of both lungs (shown by the red arrow). **(H,I)** CT images of the lung after 2 cycles of treatment showed decreased lung metastasis (shown by the red arrow compared with initial diagnosis).

Consequently, she was admitted to our hospital’s breast center for further evaluation. The general condition of the patient was fair including consciousness state, blood pressure, heart rate, respiratory rate, and normal auscultatory findings of heart and lungs. Breast ultrasound examination revealed a hypoechoic mass in the outer quadrant of the right breast, measuring 2.21*1.92 cm, displaying an indistinct border and irregular margins, graded as Breast imaging-reporting and data system (BI-RADS) category 4C. The ultrasound examination of the right axillary lymph nodes revealed the presence of multiple hypoechoic nodules with indistinct boundaries and absence of lymphatic structure **(** Figure 1C). Subsequently, a biopsy of the breast mass was performed and H&E pathological image showed the tumor cells grow in a nest, with obvious cell specificity, nuclear division and significant cell keratosis, indicated the presence of invasive carcinoma with squamous differentiation (Figures 2A). Immunohistochemistry further revealed negative expression of ER and PR, strongly positive expression of HER2, positive expression of CK7, partial positive expression of P63, membrane positive expression of P120, negative expression of E-cadherin, wild type expression of P53, and approximately 30% positive expression of Ki-67 (Figures 2B–J). Based on the integration of clinical and pathological findings, the diagnosis of MpBC and breast SCC was established. A lung computed tomography (CT) scan revealed the presence of multiple nodules with irregular morphology and burr change in both lungs, suggesting the possibility of metastatic tumor (Figures 3A, B). However, subsequent examinations including abdominal ultrasound, abdominal enhanced CT, brain magnetic resonance imaging (MRI), vertebral CT, and supraclavicular lymph node ultrasound did not reveal any signs of tumor metastasis. A positron emission tomography-computed tomography (PET-CT) examination demonstrated an increased uptake of fluorodeoxyglucose (FDG) in right chest wall soft tissue, right axillary lymph nodes, and multiple parts of both lungs, considering lymph node and multiple lung metastasis (Figures 3C–G). In conjunction with the aforementioned examination findings, the patient received a diagnosis of right breast SCC, cT4N2M1, Stage IV, HER2-positive subtype.

**FIGURE 4:**
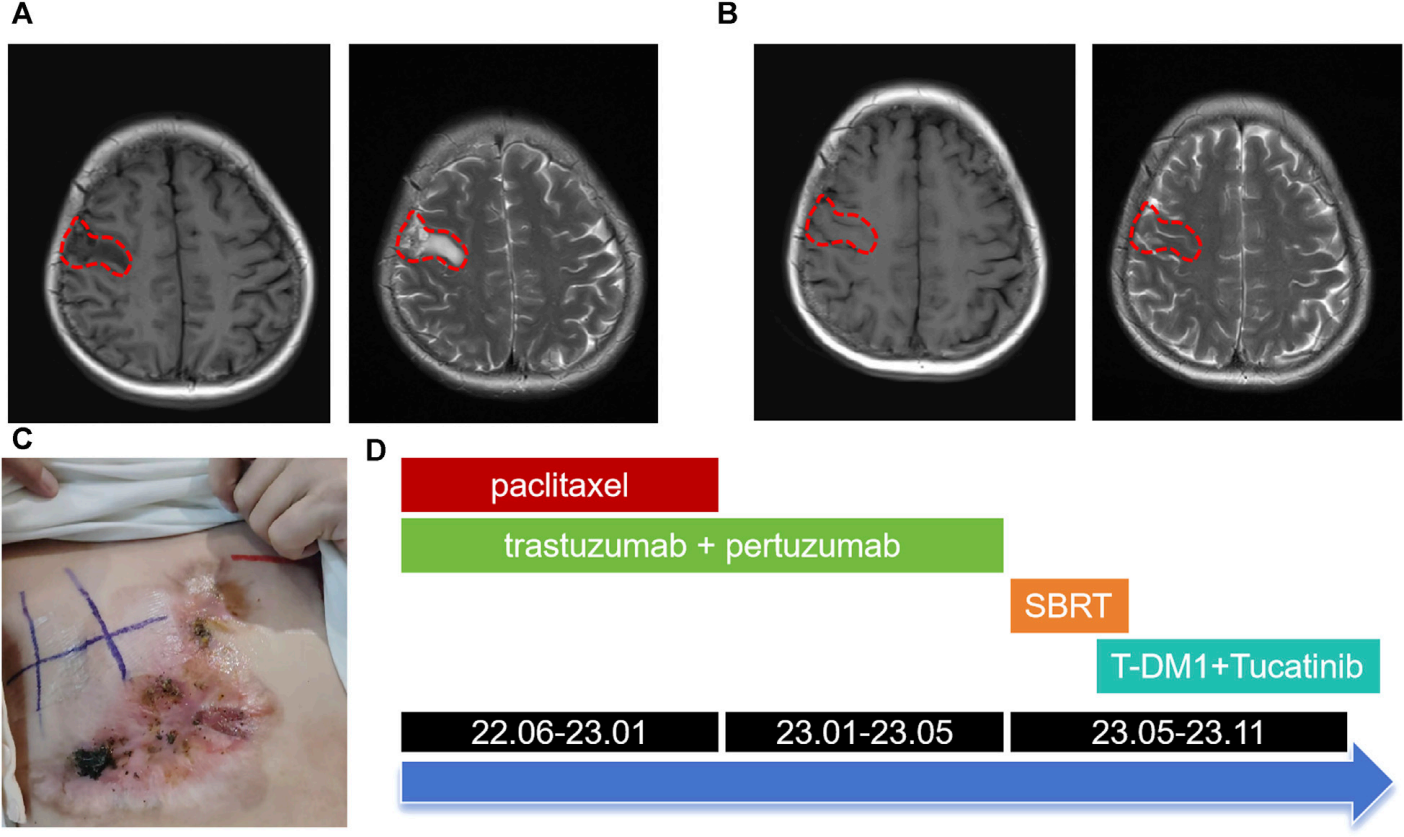
Brain MRI images and the overall treatment timeline. **(A)** Brain MRI images at May 2023 showed right frontal lobe nodule accompanied by edema in the adjacent brain tissue (lesions outlined with red dashed lines). **(B)** Brain MRI images after SBRT and combined therapy including T-DM1 combined with Tucatinib. **(C)** Changes in the right chest mass appearance at October 2023. **(D)** Treatment timeline from June 2022 to November 2023.

Following a Multi-Disciplinary Team (MDT) consultation, a treatment regimen consisting of weekly chemotherapy with paclitaxel (albumin-bound formulation) and targeted therapy with trastuzumab and pertuzumab every 3 weeks was initiated on 25 June 2022. After 6 weeks of treatment, the patient exhibited significant wound reduction (Figures 1D) and a notable decrease in lung lesions on 18 August 2022 (Figures 3H, I), which was classified as a partial response. At the same time, the evaluation of the patient’s physical condition showed that she still had a good mental state and stable vital signs, and no significant side reactions were described. Therefore, the original treatment regimen was maintained until January 2023, at which point the patient experienced bilateral lower limb edema with a normal levels of serum protein and creatinine and numbness at the end of the limb (glove anesthesia), which was attributed to paclitaxel. Consequently, paclitaxel was discontinued and the targeted therapy was continued. During the routine systematic evaluation in May 2023, a notable reduction in the right chest ulceration and scab formation on the skin were observed. Additionally, lung CT scan revealed a substantial decrease in lung metastases. However, brain MRI revealed the presence of a right frontal lobe nodule accompanied by edema in the adjacent brain tissue, and metastasis was considered (Figures 4A). Following an MDT discussion, Stereotactic Body Radiotherapy (SBRT) was administered at a dose of 32.5Gy/5F to the planning gross tumor volume (PGTV). Simultaneously, the patient’s treatment regimen was modified to include the antibody drug conjugate (ADC) T-DM1 and the tyrosine kinase inhibitor (TKI) Tucatinib, starting from 18 August 2023. Subsequent brain MRI conducted on 26 September 2023, revealed a significant reduction in the size of the brain metastasis compared to previous scans (Figures 4B). During the regimen, the patient had fatigue and platelet reduction (platelet count minimum to lower than 50*10^9/L), which may caused by the simultaneous administration of T-DM1 and radiotherapy as reported before (von Minckwitz et al., 2019). Furthermore, the patient was given daily injection of recombinant human thrombopoietin, until platelet count rose to 80*10^9/L. The patient is presently undergoing ongoing treatment, as depicted in Figures 4C, D.

## Discussion

This paper presents a comprehensive analysis of the management of an advanced case of HER2-positive breast SCC, yielding promising outcomes in targeted therapy. The findings of this study offer valuable insights for future clinical interventions in similar cases.

The etiology of MpBC remains uncertain, predominantly affecting women aged over 50 years, with sporadic occurrences and minimal familial clustering (Chaudhary et al., 2023). Limited data exists regarding the demographics, clinical manifestations, tumor attributes, and therapeutic approaches for MpBC, including SCC (Ullah et al., 2023; Wu et al., 2023). The lymph node metastasis rate of MpBC was relatively low, as evidenced by a large-scale study indicating that the incidence of MpBC involving axillary lymph nodes was 21.9%, which is lower than that of invasive ductal carcinoma (IDC) at 34.3% (Pezzi et al., 2007; Lee et al., 2023). Despite this lower lymph node involvement, MpBC is characterized by a higher propensity for local recurrence and distant metastasis, with the lung being the most common site of metastasis (He et al., 2019; Abada et al., 2022).

The majority of MpBC cases present as palpable breast masses, which do not differ from common benign and malignant breast tumors. X-ray and ultrasound are commonly advised for the diagnosis of MpBC. However, the histological complexity of MpBC makes preoperative puncture diagnosis challenging, leading to a higher reliance on postoperative diagnosis. The differential diagnosis of MpBC necessitates the utilization of multiple immunohistochemical markers. The morphological characteristics of SCC are generally distinctive, making the diagnosis less arduous. Immunohistochemistry for breast SCC typically reveals the presence of Cytokeratin (CK) markers. Additionally, it is crucial to exclude primary SCC originating from other organs, such as the lung or skin.

Due to the limited prevalence of MpBC, there is a scarcity of prospective studies specifically focusing on this subtype. The majority of available data originates from retrospective studies, and currently, there are no established guidelines for the treatment of MpBC. Consequently, its management is predominantly approached similarly to invasive ductal carcinoma (IDC). Surgical intervention is the preferred approach for the management of SCC, and in cases of well-differentiated SCC, complete excision of the tumor may suffice. Endocrine therapy is not recommended for hormone receptor-negative cancer. With the exception of low-grade adenosquamous carcinoma and fibromatosis-like metaplastic carcinomas, MpBC is limited response to conventional chemotherapy. In the event that chemotherapy is deemed necessary, the selection of 5-fluorouracil, doxorubicin, and platinum agents can be considered. Chen et al. (2011) conducted a study involving 12 patients with MpBC, administering either single or combination chemotherapy. Among these patients, only one individual who received chemotherapy containing taxol drugs exhibited a partial response. In another separate study by Takala, 14 patients undergoing palliative chemotherapy were included, with two patients showing a partial response after being treated with amycin or capecitabine (Takala et al., 2019).

The majority of MpBC patients were found to be HER2 negative, with HER2 positivity observed only in a limited number of cases or reported in retrospective studies. Expression rate of HER2 in breast SCC has also been reported to be less than 10% (Pirot et al., 2020). The HER2 protein is classified as a member of the tyrosine protein kinase family. HER2 over-expression plays a significant role in the regulation of cellular processes, specifically cell growth and division. However, when HER2 is over-expressed, it leads to the rapid proliferation of cancer cells and tumor formation. This phenomenon is commonly observed in malignancies, particularly in breast cancer. Consequently, the use of HER2-targeted antibodies, such as trastuzumab and pastuzumab, ADC (T-DM1, T-DXd), and TKI (Pyrrotinib, tukatinib, and neratinib), has become a primary approach to address HER2 over-expression in breast cancer (Swain et al., 2023). The combination of drugs targeting the HER2 protein can effectively induce antibody-dependent cell-mediated cytotoxicity (ADCC) and activate the classical pathway of complement, thereby impeding the proliferation, division, and metastasis of tumor cells. These targeted drugs have been employed in this particular case, yielding favorable therapeutic outcomes within a specific timeframe. Lei et al. (2022) documented notable efficacy in MpBC patients with positive HER2 results following trastuzumab treatment. Further investigation is warranted to ascertain the suitability of HER2-targeted therapy for HER2-positive MpBC patients, given the limited prevalence of HER2 expression in this population. Given the resistance of MpBC to chemotherapy, it is imperative to explore novel treatment options based on the molecular tumor characteristics. Notably, MpBC cases featuring somatic gene mutations in the PI3K/AKT/mTOR signaling pathway demonstrate heightened pathway activity, which is closely linked to primary and secondary resistance to conventional breast cancer therapy (Ng et al., 2017). The high-frequency alterations observed in the PI3K/AKT/mTOR pathway suggest that the use of PI3K inhibitors or mTOR inhibitors may be a viable therapeutic option for the treatment of MpBC. A study conducted by Yang et al. (2019) in 2019 demonstrated that a MpBC patient with a *PIK3CA H1047R* mutation achieved a partial response when treated with weekly paclitaxel therapy and the PI3K inhibitor Bupanisi, resulting in an overall survival of 42 months. Additionally, mTOR inhibitors have shown promise in MpBC patients. A systemic treatment regimen for advanced MpBC patients, consisting of an mTOR inhibitor (sirolimus or everolimus) in combination with liposomal doxorubicin and bevacizumab, has demonstrated efficacy. The objective response rate (ORR) was found to be 21%, with 8% achieving a complete response and 13% achieving a partial response (Dwyer and Clark, 2015). Clinical studies are needed to confirm the potential of PI3K inhibitors and mTOR inhibitors as new treatment options for MpBC patients. Two randomized phase 3 trials, OlympiAD (He et al., 2019) and EMBRACA (Romero, 2018), have demonstrated that PARP inhibitors (PARPi) are more effective than chemotherapy in patients with locally advanced/metastatic breast cancer and BRCA mutations. An additional case of MpBC patients carrying the BRCA2 mutation achieved a pathological complete response (pCR) after 6 months of neoadjuvant treatment with Trapappanib alone, indicating that PARP inhibition may be a potential therapeutic strategy for MpBC (Litton et al., 2020). Additionally, the use of PD-L1/PD-1 immune checkpoint blockade therapy shows promise. Suzanne’s study revealed that PD-L1 expression was positive in tumor cells in 18% of the enrolled high-grade MpBC cases and in tumor-infiltrating immune cells in 40% of the cases (Chartier et al., 2023). Sylvia and Anita separately reported a case of pCR achieved through a combination of chemotherapy and Pembrolizumab in MpBC (Adams, 2017; Gul et al., 2023).

## Conclusion

This paper presents a unique instance of HER2-positive breast SCC that exhibited favorable outcomes upon the administration of HER2-targeted drugs in combination with chemotherapeutic drugs. These findings suggest that HER2-targeted drugs can also yield positive effects on MpBC and SCC, thereby offering a novel point of reference for the subsequent treatment of uncommon pathological subtypes of breast cancer.

## Data Availability

The raw data supporting the conclusions of this article will be made available by the authors, without undue reservation.

## Abbreviations

ADC, Antibody Drug Conjugate; ADCC, Antibody-dependent cell-mediated cytotoxicity; BI-RADS, Breast Imaging Reporting and Data System; ER, Estrogen Receptor; HER2, Human Epidermal Growth Factor Receptor 2; IDC, Invasive ductal carcinoma; MDT, Multi-Disciplinary Team; MpBC, Metaplastic Breast Cancer; MRI, Magnetic Resonance Imaging; pCR, pathological Complete Response; PR, Progesterone Receptor; SCC, Squamous Cell Carcinoma; TKI, Tyrosine Kinase Inhibitor; TNBC, Triple-Negative Breast Cancer; WHO, World Health Organization.
